# GnRH-a-Induced Perimenopausal Rat Modeling and Black Cohosh Preparations’ Effect on Rat’s Reproductive Endocrine

**DOI:** 10.3389/fendo.2021.683552

**Published:** 2021-12-24

**Authors:** Jiming Chen, Huihui Wang, Zhiyong Dong, Junling Liu, Zhenyue Qin, Mingyue Bao, Hongxia Yu, Shoufeng Zhang, Wendi Zhang, Chunjian Qi, Jie Wu

**Affiliations:** ^1^ Department of Obstetrics and Gynecology, The Affiliated Changzhou No. 2 People’s Hospital of Nanjing Medical University, Changzhou, China; ^2^ State Key Laboratory of Reproductive Medicine, Nanjing Medical University, Nanjing, China; ^3^ Department of Obstetrics and Gynecology, The First Affiliated Hospital of Nanjing Medical University, Nanjing, China

**Keywords:** isopropanolic extract of cimicifuga racemosa, gonadotropin-releasing hormone agonist, estradiol, rat model of perimenopausal syndrome, reproductive endocrine

## Abstract

**Background:**

Endometriosis (EMS) is an estrogen-dependent disease, which easily recurs after operation. Gonadotropin-releasing hormone agonist (GnRH-a), an estrogen-inhibiting drug, can effectively inhibit the secretion of gonadotropin by pituitary gland, so as to significantly decrease the ovarian hormone level and facilitate the atrophy of ectopic endometrium, playing a positive role in preventing postoperative recurrence. The application of GnRH-a can lead to the secondary low estrogen symptoms, namely the perimenopausal symptoms, and is a main reason for patients to give up further treatment. The add-back therapy based on sex hormones can well address the perimenopausal symptoms, but long-term use of hormones may cause the recurrence of EMS, as well as liver function damage, venous embolism, breast cancer and other risks, which has long been a heated topic in the industry. Therefore, it is necessary to find effective and safe anti-additive drugs soon. Studies at home and abroad show that, as a plant extract, isopropanolic extract of cimicifuga racemosa (ICR) can well relieve the perimenopausal symptoms caused by natural menopause. Some studies have preliminarily confirmed that black cohosh preparations can antagonize perimenopausal symptoms of EMS patients treated with GnRH-a after operation.

**Objective:**

To establish a rat model of perimenopausal symptoms induced by GnRH-a injection, for the purposes of laying a foundation for further research and preliminarily exploring the effect of black cohosh preparations on reproductive endocrine of the rat model.

**Method:**

The rat model of perimenopausal symptoms was established by GnRH-a injection, and normal saline (NS injection) was used as the control. The rats were randomly divided into four groups according to different modeling methods and drug intervention schemes. GnRH-a injection + normal saline intervention group (GnRH-a + NS), normal saline injection control + normal saline intervention group (NS + NS), GnRH-a injection + estradiol intervention group (GnRH-a + E2), and GnRH-a injection + black cohosh preparations intervention group (GnRH-a + ICR). After modelling was assessed to be successful with the vaginal smear method, the corresponding drugs were given for intervention for 28d. In the process of rat modeling and drug intervention, the skin temperature and anus temperature of the rat tails were measured every other day, the body weights of the rats were measured every other day, and the dosage was adjusted according to the body weight. After the intervention was over, the serum sex hormone level, the uterine weight, the uterine index, and the endometrial histomorphology changes, as well as the ovarian weight, the ovarian index, and the morphological changes of ovarian tissues of each group were measured.

**Results:**

(1) The vaginal cell smears of the control group (NS + NS) showed estrous cycle changes, while other model rats had no estrous cycle of vaginal cells. (2) The body weight gains of the GnRH-a + NS, GnRH-a + E2 and GnRH-a + ICR groups were significantly higher than that of the NS + NS control group. The intervention with E2 and ICR could delay the weight gain trend of rats induced by GnRH-A. (3) After GnRH-a injection, the temperature of the tail and anus of rats showed an overall upward trend, and the intervention with E2 and ICR could effectively improve such temperature change. (4) The E2, FSH, and LH levels in the GnRH-a + NS, GnRH-a + E2, and GnRH-a + ICR groups were significantly lower than those in the NS + NS group (P < 0.01). The E2 level was significantly higher and the LH level was significantly lower in the GnRH-a + E2 group than those in the GnRH-a + NS and GnRH-a + ICR groups (P < 0.05). Compared with those of the GnRH-a + NS and GnRH-a + ICR groups, the FSH level of the GnRH-a + E2 group showed a slight downward trend, but the difference was not statistically significant (P > 0.05). There was no significant difference in the levels of sex hormones between the GnRH-a + NS group and GnRH-a + ICR group (P > 0.05). (5) Compared with those of the NS + NS group, the uterine weight and uterine index of the GnRH-a + NS, GnRH-a + E2 and GnRH-a + ICR groups significantly decreased (P < 0.01). In a comparison between the groups, the uterine weight and uterine index in the GnRH-a + NS and GnRH-a + ICR groups were significantly lower than those in the GnRH-a + E2 group (P < 0.01). There was a statistical difference in the uterine weight and uterine index between the GnRH-a + NS group and GnRH-a + ICR group (P > 0.05). (6) Compared with those of the NS + NS group, the ovarian weight and ovarian index of the GnRH-a + NS, GnRH-a + E2 and GnRH-a + ICR groups significantly decreased (P < 0.01). There was no statistical difference in the ovarian weight and ovarian index among the GnRH-a + E2, GnRH-a + NS and GnRH-a + ICR groups (P > 0.05). (7) Compared with those in the NS + NS group, the number of primordial follicles increased significantly, while the number of growing follicles and mature follicles decreased significantly in the GnRH-a + NS, GnRH-a + E2, and GnRH-a + ICR groups (P < 0.01), but there was a statistical difference in the total number of follicles among the four groups (P > 0.05).

**Conclusions:**

The GnRH-a injection could achieve the desired effect. The animal model successfully achieved a significant decrease in the E2, FSH, and LH levels in rats, and could cause the rats to have rising body surface temperature similar to hot flashes in the perimenopausal period. The intervention with E2 and ICR could effectively relieve such “perimenopausal symptoms”, and ICR had no obvious effect on the serum sex hormone level in rats.

## Introduction

Endometriosis, EMS for short, means that functional endometrial glands or stromata are ectopic outside the uterine cavity, which mainly occurs in female genitalia, peritoneum, bladder and rectum. It is a common gynecological benign disease, which occurs in about 10%-15% of women of childbearing age (1), and the incidence rate has obviously increased in recent years (2). Clinically, EMS is characterized by dysmenorrhea, chronic pelvic pain, dyspareunia, abnormal menstruation, infertility (in over 50% of women), etc. (3) EMS seriously affects the quality of life and reproductive health of female patients, which is one of the most troublesome problems for gynecologists. EMS has a wide range of pathological changes and forms. It is a benign lesion, but has malignant biological behavior such as infiltration, metastasis, and recurrence (4). For a long time, this disease, which is benign in pathology but with biological behavior similar to malignant lesions, has been besetting clinicians and patients. There are many methods to diagnose and treat EMS at present, but the results are not satisfactory. This is because no matter what treatment is used, the recurrence rate of EMS within 5 years after treatment is over 40% (5). Currently a consensus is that the main purpose of treatment of EMS is to eliminate lesions, relieve pain, solve the fertility problem, and reduce recurrence (6). Surgical treatment is one of the main treatments of EMS. 75% of EMS cases are mild or minor lesions, and 25% are moderate to severe lesions. Currently surgical treatment is applied to patients with moderate to severe lesions, and some patients with mild or minor lesions also have surgical indications, especially for laparoscopic surgery (7). As a sex-hormone-dependent disease, EMS often occurs to women of childbearing age. The effect of EMS progression on fertility and the recurrence of such disease cannot be ignored. The biological basis of EMS recurrence is the survival of ectopic endometrial cells which are maintained by corresponding hormones. Thoroughly stripping or excising endometriosis is a key to improving the treatment effect and reducing postoperative recurrence. Currently, laparoscopic surgery is the best operation method for EMS, the inhibition of the ovarian function is the best drug therapy for EMS, and laparoscopic surgery + drug therapy is the best combined therapy for EMS. Patients with EMS undergo conservative surgery to preserve their reproductive function, and it is often impossible to completely remove ectopic endometrial lesions during the operation. The residual ectopic endometrium always has the metabolic activity and regrowth potential.

## Materials and Methods

### Materials

#### Experimental Animals

Twenty-four female Sprague-Dawley (SD) clean rats, aged 5 - 6 weeks and weighing (200 ± 20)g, were purchased from Slac Laboratory Animal, and were approved by the Experimental Animal Welfare Ethics Committee. License No. for experimental animals: SCXK (Hu) 2012-0002; 6 rats/fed in polycarbonate cage with sufficient food and water. The rats were free to drink and eat during the trial. The rats were fed with granular feed without soybean until the end of the trial, so as to remove the influence of phytoestrogen on the trial result. The rats were kept at room temperature of (25 ± 1)°C, relative humidity of 50% - 55% and circadian rhythm of 12 hours. After the rats were adapted to the feeding environment for one week, the trial officially started. In this study, all experimental operations on the experimental animals were carried out by personnel with experimental animal work certificates. All experimental operations complied with the *Regulations on Experimental Animals of the People’s Republic of China* promulgated by the State Scientific and Technological Commission of China and the *Regulations on Laboratory Animals of Jiangsu Province.*

#### Investigational Drugs and Reagents

(1) Black cohosh preparations (Schaper&Briimmer GmbH & Co. KG, Germany). (2) Estradiol valerate (Guangzhou Branch of Bayer Healthcare Co., Ltd.), trade name Progynova, 1mg/tablet. (3) Gonadotropin releasing hormone agonist (GnRH-a): (Beaufour Ipsen France), 3.75mg/injection. (4) 0.9% saline for injection (0.9% saline, NS): (Zhejiang Jimin Pharmaceutical Factory). (5) 10% chloral hydrate: 10g of chloral hydrate was dissolved in a proper amount of physiological saline, the volume was fixed to 100ml, and the mixture was fully stirred and mixed with an electromagnetic stirrer, so that the 10% concentration required for injection was prepared.

#### Enzyme Linked Immunosorbent Assay (ELISA) Reagents

(1) E2ELISA detection kit (Uscnk, item number: CEA461Ge, manufacturing batch number: L140616347); (2) FSH ELISA detection kit (Uscnk, item number: CEA830Ra, manufacturing batch number: L140619501); (3) LHELISA detection kit (Uscnk, item number: CEA441Ra, manufacturing batch number: L140619417); (4) Other reagents: distilled water, etc.

#### HE Staining Experiment Reagent

(1) Xylene (Chengdu Kelong Chemical Reagent Factory); (2) Absolute ethanol (Chengdu Kelong Chemical Reagent Factory); (3) Hematoxylin (SIGMA, batch number: 041M0014V); (4) Eosin (Shanghai Maikun Chemical Co., Ltd., batch number: 20120831); (5) Hydrochloric acid solution (Hangzhou Shuanglin Chemical Reagent Factory); (6) Neutral balsam (Shanghai Specimen Model Factory).

#### Main Instruments and Equipment

(1) Ultra-low temperature refrigerator (BS-812); (2) High-speed freezing centrifuge (Hunan Xiangyi Equipment, model TGL-16m); (3) Stereotaxic apparatus (Chengdu Taimeng Technology Co., Ltd., model DW-2000); (4) Electronic thermometer (Hangzhou Yisida Co., Ltd., measuring range 0 - 100°C); (5) Electronic balance (MettlerToledo, Switzerland, model GB204); (6) Full-wavelength microplate reader (USA MD, model SpectraPlus 384); (7) Incubator (Shanghai Jinghong, model DHG-9070A); (8) Micropipettor; (9) Rotary slicer (LEICA, RM2235); (10) Pathological tissue drier (Changzhou Haosilin Instrument and Equipment Co., Ltd., tec2500); AD microscope (OLYMPUS, BX43); (11) Water-jacket constant temperature incubator (Shanghai Yuejin Medical Equipment Co., Ltd., PYX-DHS500BS-II); (12) Multifunctional infrared digital thermometer (SHARP, Japan).

### Methods

#### Animal Modelling

(1) Animal modelling by drug castration with GnRH-a injection: GnRH-a (Triptorelin Acetate for Injection) was injected intramuscularly for 7 days to SD adult female rats, and then maintained at 1/5 of the initial dose every day. The dosage of GnRH-A was the routine clinical dosage of 0.05 mg/kg, and the maintenance dosage of 0.01mg/kg, which was obtained after conversion according to the body surface area of humans and rats (8–10) from the human standard weight to animal non-standard weight:

Db = Da.Rab.Sb (11).

(2) Saline injection control group: Normal saline was injected intramuscularly for 7 days to SD adult female rats, and then maintained at 1/5 of the initial dose every day. The purpose of setting up the normal saline control group was to remove the confounding factors in the trial result caused by drug injection.

#### Analysis of Vaginal Smears in Rats

To test whether modelling with the GnRH-a injection was successful, vaginal smears were done once every day two weeks after GnRH-a injection (intramuscular injection at 0.06mg/kg for one week, intramuscular injection at 0.012mg/kg for one week) to observe the modeling effect. The vaginal smear analytical method was used for examination. A dropper filled with 0.9% normal saline was gently inserted into the vagina of the rat by about 1-2cm, and normal saline was injected and then sucked out, which was repeated twice to three times. The vaginal irrigation solution in the dropper was dripped on the glass slide. The number of semitransparent flat epidermal cells in the liquid was observed under the inverted height electron microscope for 5 to 7 days. If the number of flat epidermal cells did not increase on the continuous observation days, it showed that GnRH-a modelling was successful; otherwise it failed, and the rat should be excluded as an experimental subject.

The estrous cycle of rats generally lasts for 4 to 5 days, which can be divided into the proestrus, estrus, metestrus, and diestrus. The vaginal smear and alkaline methylene blue staining were used to observe the cell morphology under the light microscope from 8:00 to 10:00, to judge the different stages of the estrous cycle. The judgment details are as follows. Proestrus: The vaginal smear showed that most of them were nucleated epithelial cells, and a small number of keratinized epithelial cells were occasionally seen. Estrus: Most of the enucleated keratinized epithelial cells accumulated in a deciduous shape, and a small number of epithelial cells were occasionally seen. Metestrus: The vaginal smears of rats showed leukocytes, keratinocytes, and nucleated epithelial cells. Diestrus: There were a large number of white blood cells, and a small number of epithelial cells and mucous cells in the vaginal smears of rats.

#### Preparation of Dosage Form and Intragastric Administration

All rats were treated with the drug after modelling was identified as successful (4th week after GnRH-a injection). The investigational drug was prepared as follows: Estradiol valerate (E2) and black cohosh preparations (1CR) tablets were dissolved in sterile physiological saline after ultrasonic treatment to form a uniform turbid liquid (12–14). The preparations concentration of E2 was 0.2g/L. The preparations concentration of ICR was 12g/L (expressed as the concentration of crude drugs. Every tablet of black cohosh preparations contained 20mg crude drugs). The drug was administered by gavage according to the following dosage at 8:00 a.m. and 9:00 a.m. every day. GnRH-a + NS group: intragastric administration of normal saline, 10mL/kg; NS + NS group: intragastric administration of normal saline, 10mL/kg; GnRH-a + E2 group: intragastric administration of E2, 0.8mg/kg; GnRH-a + ICR group: intragastric administration of ICR, 60mg/kg (based on crude drug). The rats were weighed once every other day, and the dosage (ml) was adjusted according to the change of the body weight.

#### Specific Trial Grouping and Medication Intervention Scheme

After modelling of the experimental animals was identified as successful, the rats were randomly divided into 4 groups according to the modeling method and drug intervention scheme, with 6 rats in each group:

(1) GnRH-a injection + normal saline intervention group (GnRH-a + NS): After the GnRH-a injection modelling was identified as successful, normal saline for injection (l0mL/kg) started to be given intragastrically (10mL/kg) every day from Week 4 after GnRH-a injection, for a total of 28 days;
(2) Normal saline injection control + normal saline intervention group (NS + NS): After modelling of the NS injection control group was identified, normal saline for injection (l0mL/kg) started to be given intragastrically (10mL/kg) every day from Week 4 after NS injection, for a total of 28 days;
(3) GnRH-a injection + estradiol intervention group (GnRH-a + E2): After the GnRH-a injection modelling was identified as successful, E2 started to be given intragastrically every day (0.8mg/kg) from Week 4 after GnRH-a injection, for a total of 28 days;
(4) GnRH-a injection + black cohosh preparation intervention group (GnRH-a + ICR): After the GnRH-a injection modelling was identified as successful, ICR started to be given intragastrically every day (60mg/kg based on crude drugs) from Week 4 after GnRH-a injection, for a total of 28 days.

#### Determination of Body Weight and Body Temperature of Rats

The trial operators used an electronic balance to weigh the rats once every other day. After rat modeling and drug intervention in each group, corresponding drugs were selected for intragastric intervention according to the groups of rats. The daily dose and administration mode of drugs were based on those in literature reports for reference (12, 15). After the establishment of the model was successful, the fixed personnel finished the administration at 8: 00 am every morning. After the beginning of the experiment, the anal temperature and tail skin temperature of the rats were measured regularly by the fixed experimental operators every other day. The rectal temperature of the rats was measured by the electronic thermometer. The thermometer was inserted into the anal depth of the rat about 2.0cm, and the value was read after the duration of l.0min. The skin temperature of rat tail was measured by multi-function infrared digital thermometer. The skin temperature was measured at the distance from the 2.0cm of the root of rat tail. After 30 seconds, the corresponding values were read and the temperature data were recorded. According to the temperature data, the average temperature of each group was taken, and the corresponding “temperature-time curve” was drawn, and the body mass of rats was recorded dynamically. According to the results of body weight recording, the average weight of rats in each group was taken to draw the “body weight-time change map”.

#### Execution of Experimental Rats and Collection of Blood Samples

After drug treatment intervention, rats in each group were anesthetized intraperitoneally with 10% chloral hydrate (0.35ml/100g), and 5ml of trunk blood was collected from the abdominal vein of rats for later use. After the rat was executed, the tissue samples were taken for testing.

#### Determination of Serum Sex Hormones in Rats

After modeling and drug intervention in each group, trunk blood was collected from the abdominal vein for later use. Specifically, 2ml of the samples was sent to the laboratory, and the serum sex hormones, including estradiol (E2), follicle stimulating hormone (FSH) and luteinizing hormone (LH), were detected using the enzyme-linked immunosorbent assay (ELISA) method.

#### Hematoxylin-Eosin (He) Staining and Light Microscope Were Used to Observe the Pathological Changes of Uterine and Ovarian Tissue of Rats in Each Group

Morphological observation of ovarian tissue:

There is no peritoneum on the surface of ovary, and ovary is covered with a single layer of flat or cubic epithelium called germinal epithelium. Under the germinal epithelium is a layer of dense fibrous tissue called the tunica albuginea ovarii. Further inward is the parenchyma part of ovary, which is divided into cortex and medulla (or endoplasm). Cortex, also known as the parenchyma layer, is the main part of ovary, which is located in the outer layer. There are follicles in different developmental stages in the ovarian cortex, including primordial follicles (PF), primary follicles (PrF), secondary follicle (SF), mature follicles (MF), and atresia follicles (AF). Primary follicles and secondary follicles are collectively called growing follicles (GF). The morphological characteristics of follicles at all levels are shown in **Table 1** below.

**Table 1:** Morphological characteristics of follicles at all levels under microscope (13).

Follicle classification	Morphological characteristics under microscope
Primordial follicle	They are located in the superficial cortex, with a large number and small size, and are composed of a primary oocyte and a layer of flat follicle cells around it. The oocyte is large, with large and round nucleus, is vacuole-like and has obvious nucleoli. The boundaries of egg cells are unclear, and the nucleus is oblate.
Primary follicle	They are located in the deep cortex. The primary oocytes are slightly larger, the granulosa cells are monolayer cubic or short columnar, and the zona pellucida appears between oocytes and follicular cells.
Secondary follicle	Follicular cavities appear between follicular cells, and oocytes and surrounding follicular cells squeeze to one side of follicles to form ovarian cumulus. Corona radiata appears around oocytes.
Mature follicle Atretic follicle	Follicles increase in size and protrude to the surface of ovary.They are degenerated follicles, which can occur in all stages of follicular development, are manifested as irregular shape of oocytes, nuclear pyknosis or atrophy of oocytes, atrophy and collapse of zona pellucida. Granulocytes are loose and detached to the follicular cavity, and theca cells develop hyperplasia and hypertrophy.

#### Statistical Processing

SPSS13.0 software package was used for statistical analysis. GraphPadPrism5 software was used to draw relevant charts.

## Result

### Result Analysis of Vaginal Smear

**Figure 1:**
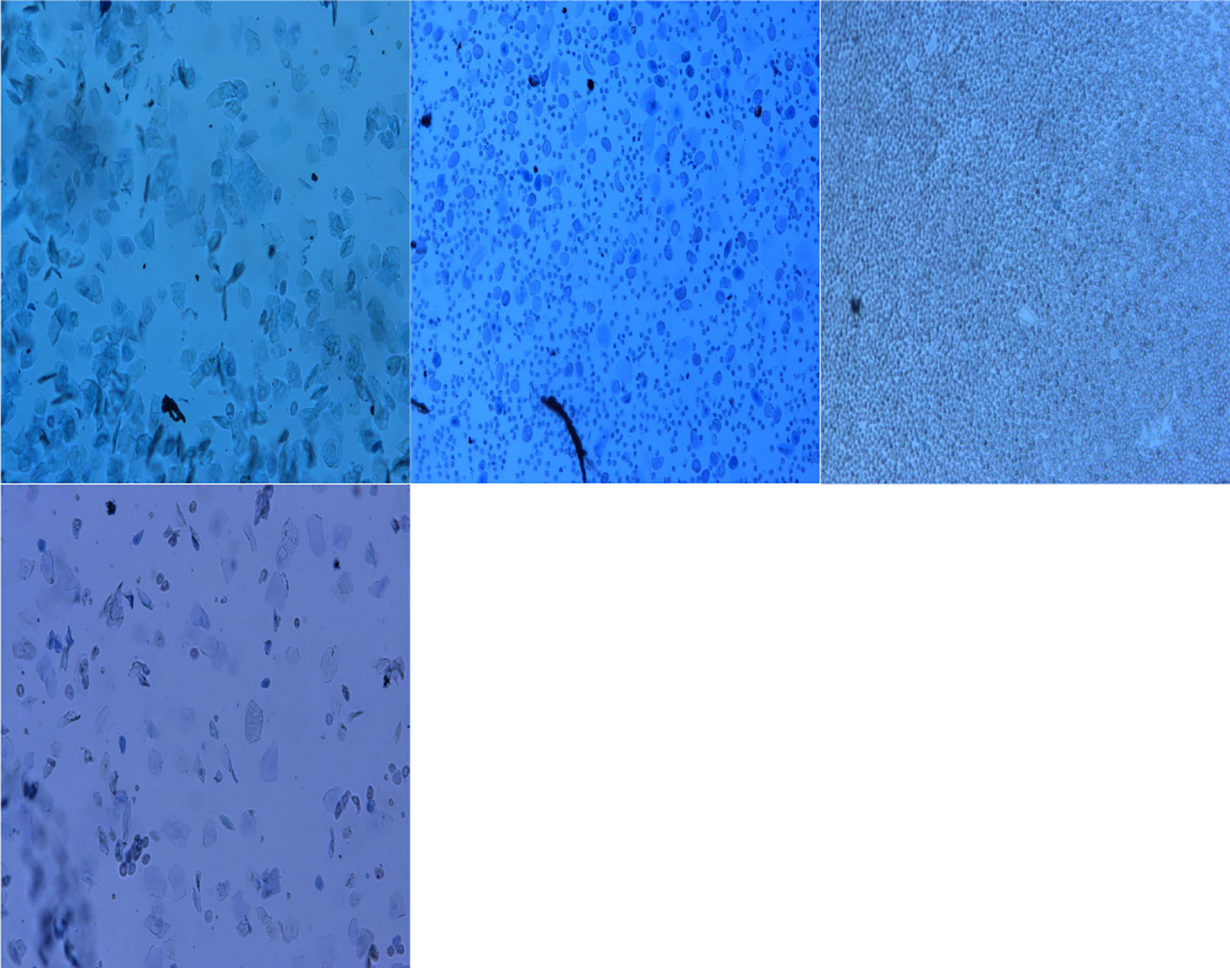
Vaginal cell smear of rats in control group (NS + NS).

**Figure 2:**
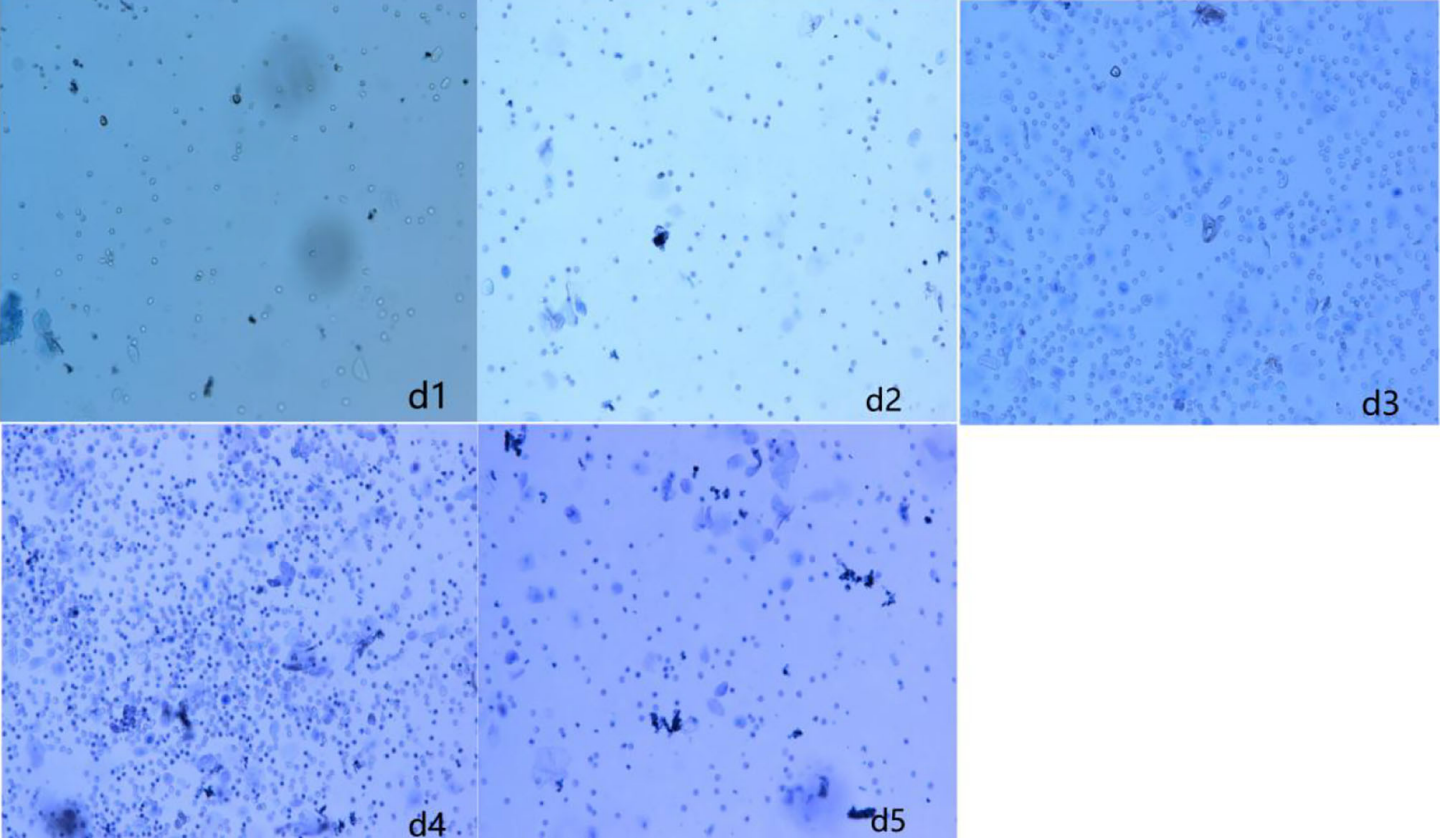
Vaginal cell smear of rats in model group (GnRH-a injection modelling). d1–d5 (first day to the fifth day of our observation).

The vaginal smear results of rats in each group showed that the vaginal smears of rats in the control group (NS + NS) showed estrous cycle changes (see **Figure 1**). The components of cell smears varied, and most of them were polygonal superficial cells with less cytoplasm. In the other model groups, the model rats had no estrous cycle, and only stayed in the diestrus. The components of cell smears were relatively simple, and the smears showed that white blood cells were dominant, with a few nucleated epithelial cells and mucous cells (see **Figure 2**).

### Body Weight Change of Rats

#### Trend of Body Weight-Time Change in Rats

**Figure 3:**
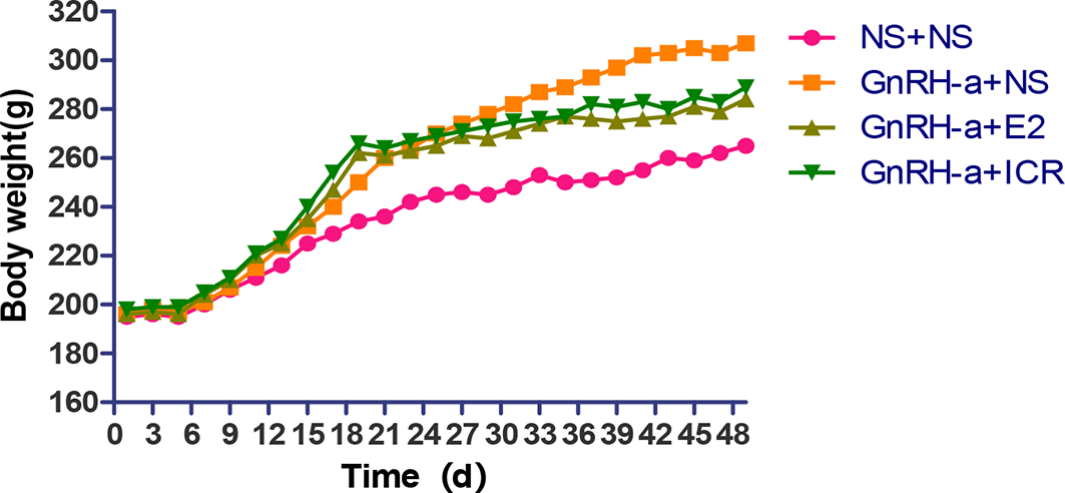
“Body weight-time change map” of rats.

In the three-week feeding period of animal modeling and identification, the body weight gain of rats in the three groups injected with GnRH-a (GnRH-a + NS, GnRH-a + E2, GnRH-a + ICR) was significantly higher than that in the control group (NS + NS). In the drug intervention period (28d), the weight gain of rats in the GnRH-a + E2 and GnRH-a + ICR groups was significantly slower than that in the GnRH-a + NS group. See **Figure 3** for details (the rats were weighed and recorded every other day, and the “weight-time change map” of rats was drawn according to the recorded results). As shown in **Figure 3**, during the three-week feeding period (121d) of animal modeling and modeling identification, the body weight of the rats of the GnRH-a model showed an obvious trend of increase, which was significantly faster than that of the control group. After drug intervention (22-49d), the trend of body weight gain of the rats in the GnRH-a + E2 and GnRH-a + ICR groups slowed down significantly. It suggested that the intervention with E2 and ICR could slow down the weight gain of rats caused by GnRH-a.

#### Comparison of Weight Changes of Rats in Each Group

**Figure 4:**
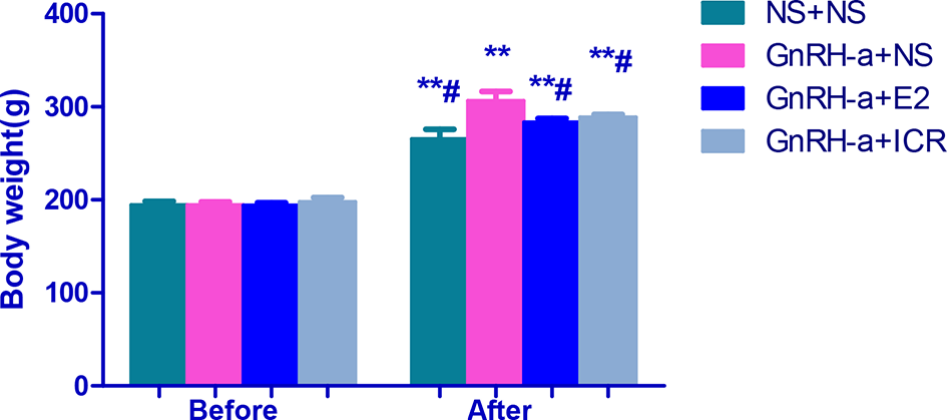
Body weight comparison of rats in each group before and after treatment intervention. **P < 0.01 vs. the same group before model establishment; ^#^P < 0.05 vs. GnRH-a + NS group after intervention. In the picture, Before = Before model establishment; After = After intervention.

There was no significant difference in body weight between the groups before modeling (P > 0.05). After intervention, the weight of rats in each group was significantly higher than that before modeling, and the difference was statistically significant (P < 0.01). After intervention, the weights of rats in each group were compared. The weights of the rats in the NS + NS, GnRH-a + E2 and GnRH-a + ICR groups after intervention were significantly lower than that of the GnRH-a + NS group (P < 0.05). The body weights of the rats in the GnRH-a + E2 and GnRH-a + ICR groups after intervention showed an upward trend compared with those in the NS + NS group, but the difference was not statistically significant (P > 0.05). See **Table 2** and **Figure 4**.

**Table 2:** Body weight changes of rats in each group before and after treatment intervention.

Group	N	Before model establishment	After intervention
NS + NS	6	194.5 ± 9.566	265.3 ± 25.97**^#^
GnRH-a + NS	6	194.5 ± 8.216	306.7 ± 24.25**
GnRH-a + E2	6	194.0 ± 6.573	283.5 ± 9.690**^#^
GnRH-a + ICR	6	197.7 ± 12.11	288.8 ± 7.195**^#^

**P < 0.01 vs. the same group before model establishment; ^#^P < 0.05 vs. GnRH-a + NS group after intervention.

### Body Temperature Changes of Rats in Each Group

#### Change Trend Analysis of Tail Skin Temperature of Rats in Each Group

**Figure 5:**
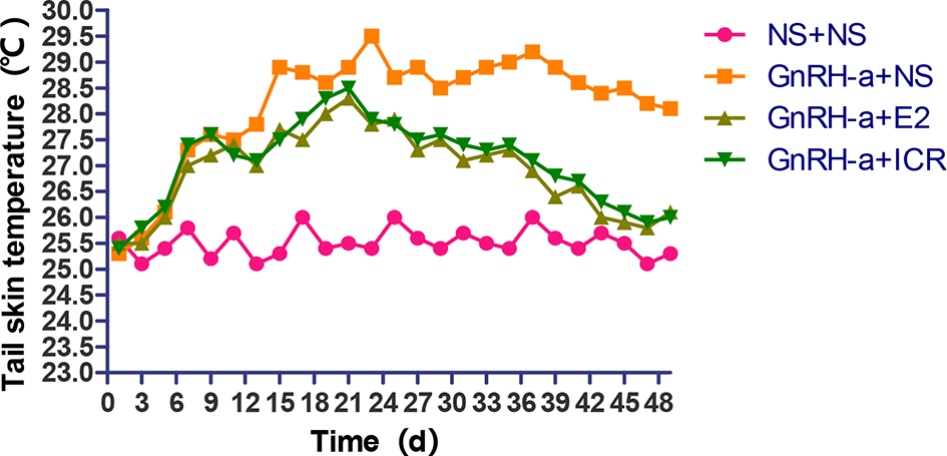
Change trend of tail skin temperature of rats in each group.

During the three-week feeding period of animal modeling and identification, the tail temperature of rats in the three groups injected with GnRH-a (GnRH-a + NS, GnRH-a + E2, GnRH-a + ICR) showed an overall upward trend. In the first 1 to 3 days of GnRH-A injection, the tail temperature of the three groups increased slowly, and did not differ greatly with that in the control group (NS + NS). From the 3rd day to the 6th day, the tail temperature of rats in the GnRH-a + NS, GnRH-a + E2, and GnRH-a + ICR groups increased significantly, which was significantly higher than that in the NS + NS group. In the drug intervention period (22-49d), the tail temperature of rats in the GnRH-a + NS group fluctuated and showed a slight downward trend. The tail temperature of the rats in the GnRH-a + E2 and GnRH-a + ICR groups decreased significantly faster than that in the GnRH-a + NS group. See **Figure 5** for details. It suggested that the GnRH-a injection could cause the rats to have rising body surface temperature similar to perimenopausal hot flashes, and the intervention with E2 and ICR could improve this situation.

#### Change Trend of Anal Temperature of Rats in Each Group

**Figure 6:**
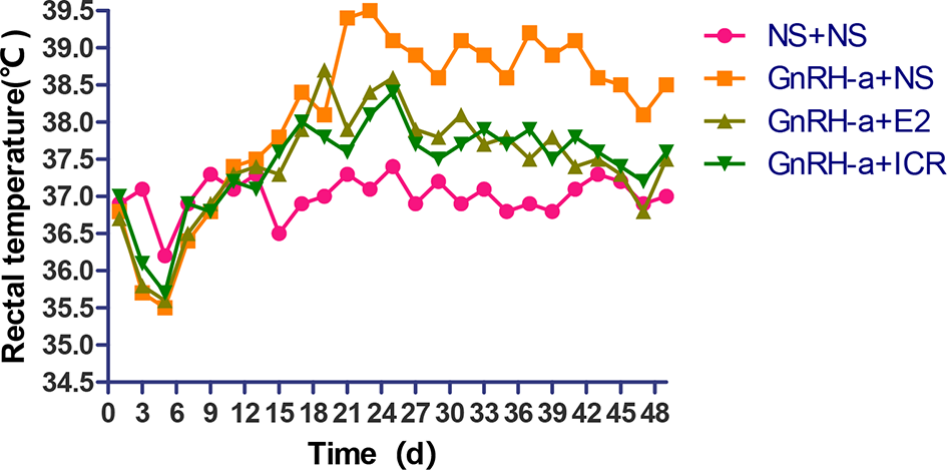
Change trend of anal temperature of rats in each group.

In the three-week feeding period of animal modeling and identification, the anal temperature of rats in the three groups injected with GnRH-a (GnRH-a + NS, GnRH-a + E2, GnRH-a + ICR) showed a drop-and-then-slow-rise trend. The anal temperature of rats in the three groups injected with GnRH-a in the first 1 to 5 days gradually decreased, which was slightly lower than that of the control group (NS + NS). The anal temperature of rats in the GnRH-a + NS, GnRH-a + E2, and GnRH-a + ICR groups increased after 5 to 6 days, but did not differ greatly from that in the NS + NS group. After 18 to 20 days, the anal temperature of the rats injected with GnRH-a in the three groups was significantly higher than that in the NS + NS group. In the drug intervention period (22-49d), the anal temperature in the GnRH-a + NS group fluctuated and showed a downward trend. The anal temperature of rats in the GnRH-a + E2 and GnRH-a + ICR groups decreased significantly faster than that in the GnRH-a + NS group. See **Figure 6** for details. It suggested that after the GnRH-a injection caused the rats to have rising body surface temperature similar to perimenopausal hot flashes, which might cause temperature fluctuations and changes *in vivo*, and E2 and ICR could relieve this situation.

### Serum Sex Hormone Levels of Rats in Each Group After Treatment Intervention

**Figure 7:**
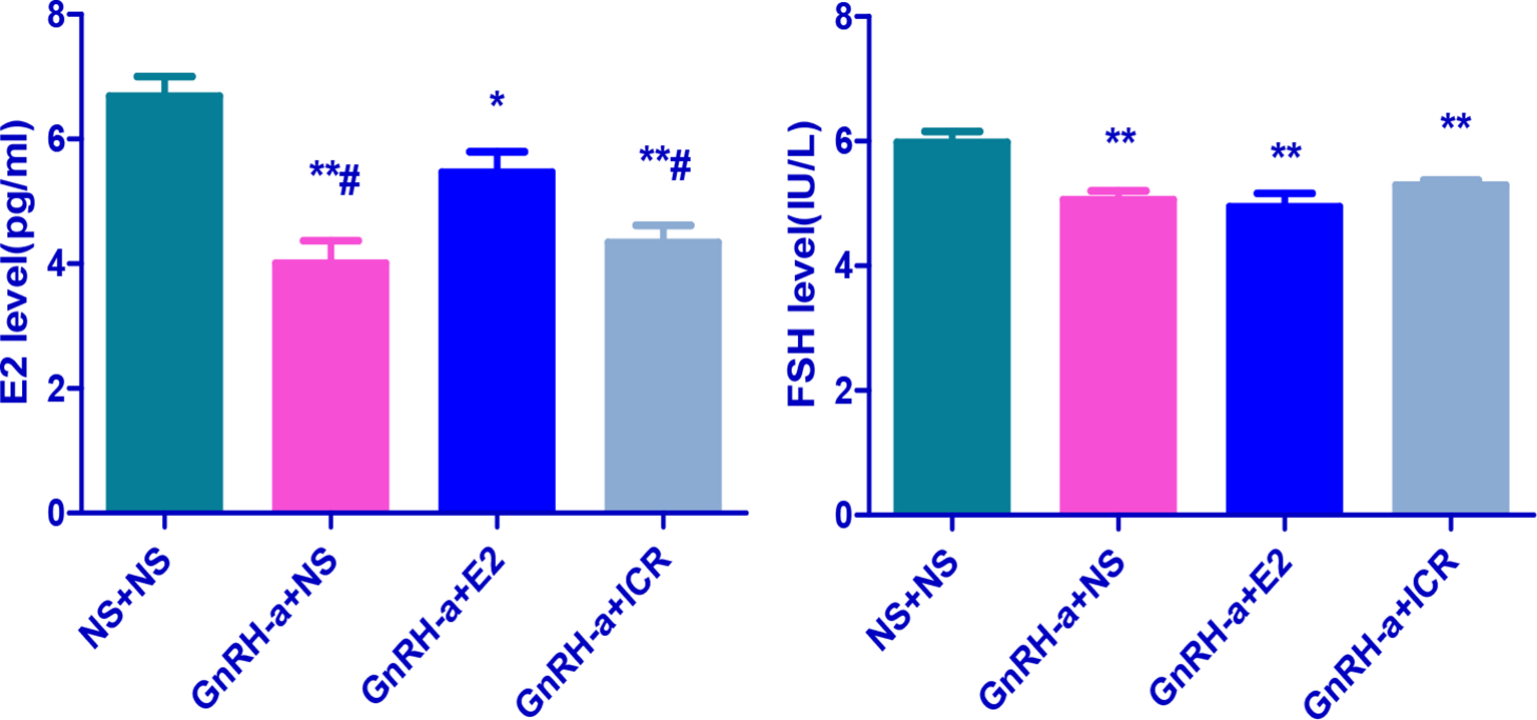
Comparison of sex hormone levels of rats in each group after treatment intervention. *P < 0.05, **P < 0.01 vs. NS + NS; ^#^P < 0.05 vs. GnRH-a + E2.

After the treatment intervention, the serum E2 levels of the GnRH-a injected rats in the three groups (GnRH-a + NS, GnRH-a + E2, GnRH-a + ICR) were significantly lower than those in the NS + NS group (P < 0.01). The E2 level in the GnRH-a + E2 group was significantly higher than that in the GnRH-a + NS and GnRH-a + ICR groups (P < 0.05). There were no statistical differences in the E2 level between the GnRH-a + NS group and GnRH-a + ICR group (P > 0.05). After the treatment intervention, the FSH levels of the GnRH-a injected rats in the three groups (GnRH-a + NS, GnRH-a + E2, GnRH-a + ICR) were all significantly lower than those in the NS + NS group (P < 0.01). Compared with those in the GnRH-a + NS and GnRH-a + ICR groups, the FSH level in the GnRH-a + E2 group decreased slightly, but the difference was not statistically significant (P > 0.05). There were no statistical differences in FSH level between the GnRH-a + NS group and the GnRH-a + ICR group (P > 0.05). After the treatment intervention, the LH levels of the GnRH-a injected rats in the three groups (GnRH-a + NS, GnRH-a + E2, GnRH-a + ICR) were all significantly lower than that in the NS + NS group (P < 0.01). The LH level in the GnRH-a + E2 group was significantly lower than those in the GnRH-a + NS and GnRH-a + ICR groups (P < 0.05). There were no statistical differences in LH level between the GnRH-a + NS group and the GnRH-a + ICR group (P > 0.05). The results showed that the GnRH-a injection could achieve the desired effect, and the animal model successfully achieved a significant decrease in the E2, FSH, and LH levels in rats. Meanwhile, the intervention with ICR had no significant effect on the serum sex hormone levels in rats. See **Table 3** and **Figure 7**.

**Table 3:** Comparison of plasma sex hormone levels of rats in each group after treatment intervention.

Group	N	E2(pg/ml)	FSH(IU/L)	LH(IU/L)
NS + NS	6	6.69 ± 0.77	5.98 ± 0.43	56.15 ± 3.91
GnRH-a + NS	6	4.01 ± 0.88**^#^	5.06 ± 0.33**	49.53 ± 6.09*^#^
GnRH-a + E2	6	5.47 ± 0.79*	4.95 ± 0.51**	43.43 ± 1.75**
GnRH-a + ICR	6	4.34 ± 0.68**^#^	5.29 ± 0.19**	48.94 ± 3.49*

*P < 0.05, **P < 0.01 vs. NS + NS; ^#^P < 0.05 vs. GnRH-a + E2.

### Uterine Weight, Uterine Index, and Morphological Changes of Endometrium Tissues of Rats in Each Group After Treatment

#### Comparison of Uterine Weight and Uterine Index of Rats in Each Group After Treatment

**Figure 8:**
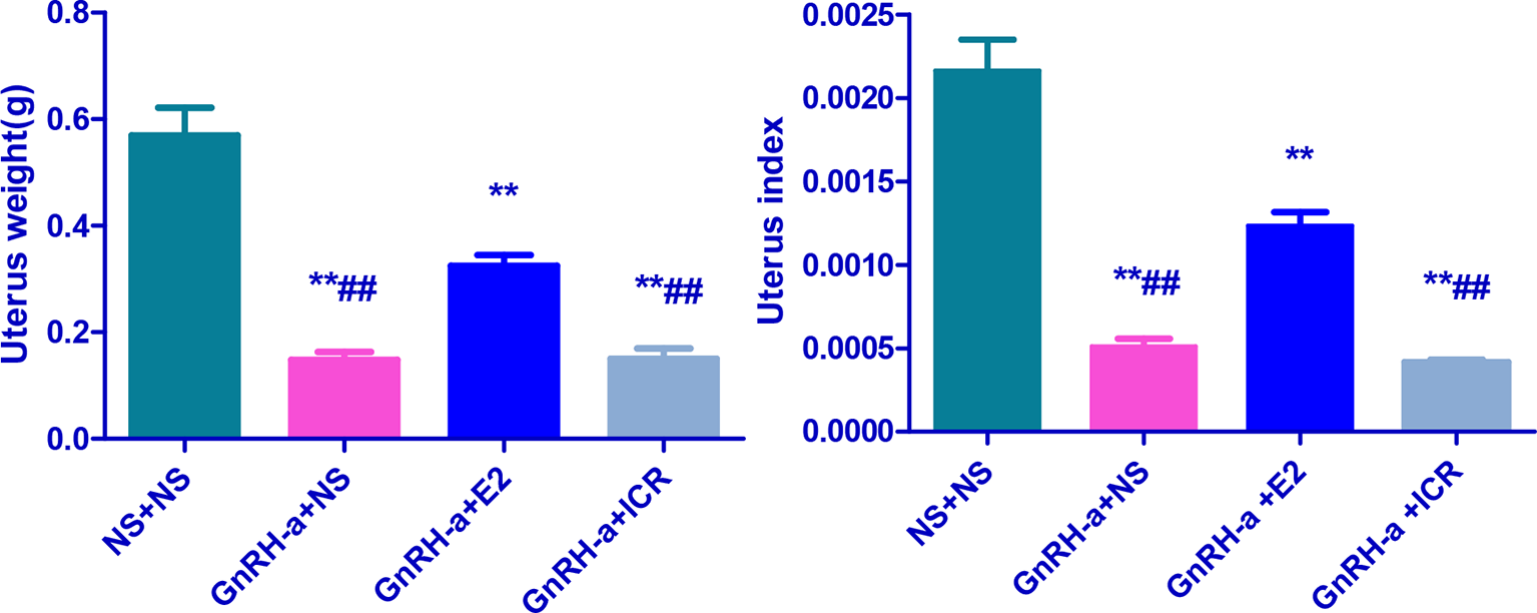
Comparison of uterine weight and uterine index of rats in each group after treatment. **P < 0.01 vs. NS + NS; ^##^P < 0.01 vs. GnRH-a + E2.

The uterine weights of the NS + NS, GnRH-a + NS, GnRH-a + E2, and GnRH-a + ICR groups were (0.48-0.56)g, (0.12-0.21)g, (0.29 - 0.36)g, and (0.11 - 0.22)g, respectively. The uterine indexes of rats in each group were (0.002164 ± 0.0004516), (0.0005123 ± 0.0001094), (0.001234 ± 0.0002007), and (0.0004177 ± 0.00001531), respectively. Compared with those in the NS + NS group, the uterine weight and uterine index in the GnRH-a + NS, GnRH-a + E2, and GnRH-a + ICR groups significantly decreased (P < 0.01). In the pairwise comparison between the groups, the uterine weight and uterine index in the GnRH-a + NS and GnRH-a + ICR groups were significantly lower than those in the GnRH-a + E2 group (P < 0.01). There was no statistical difference in uterine weight and uterine index between the GnRH-a + NS group and the GnRH-a + ICR group (P > 0.05). See **Table 4** and **Figure 8**.

**Table 4:** Comparison of uterine weight and uterine index of rats in each group after treatment.

Group	N	Uterus wet weight (g)	Uterus index
NS + NS	6	0.5700 ± 0.1256	0.002164 ± 0.0004516
GnRH-a + NS	6	0.1483 ± 0.03545**^##^	0.0005123 ± 0.0001094**^##^
GnRH-a + E2	6	0.3250 ± 0.04970**	0.001234 ± 0.0002007**
GnRH-a + ICR	6	0.1500 ± 0.04817**^##^	0.0004177 ± 0.00001531**^##^

**P < 0.01 vs. NS + NS; ^##^P < 0.01 vs. GnRH-a + E2.

#### Morphological Changes of Endometrial Tissues After Treatment

**Figure 9:**
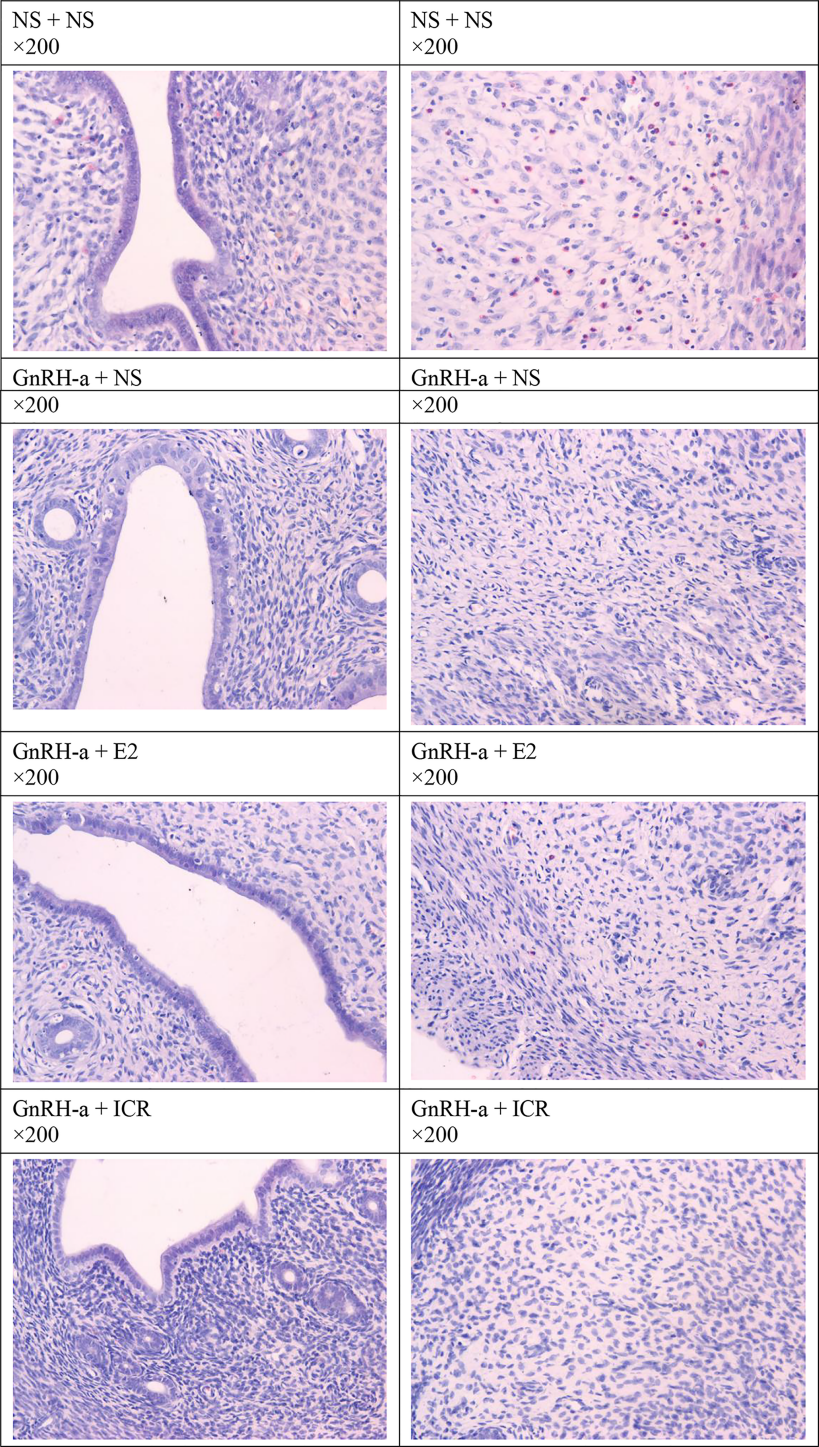
Morphological changes of endometrial tissue of rats in each group after treatment.

The endometrial tissues of rats in each group were pathologically sectioned, stained with HE, and the morphological changes of endometrial tissues of each group were observed under microscope. The results showed that in the NS + NS group, the inner membrane structure was normal, and there were many eosinophils in the interstitium. In the GnRH-a + NS group, the inner membrane structure was normal, and eosinophils were not visible. In the GnRH-a + E2 group, eosinophils decreased in the intimal epithelium. In the GnRH-a + ICR group, the epithelial structure was normal, and eosinophils were not visible. See **Figure 9**.

### Ovarian Weight, Ovarian Index, and Morphological Changes of Ovarian Tissues of Rats in Each Group After Treatment

#### Comparison of Ovarian Weight and Ovarian Index of Rats in Each Group After Treatment

**Figure 10:**
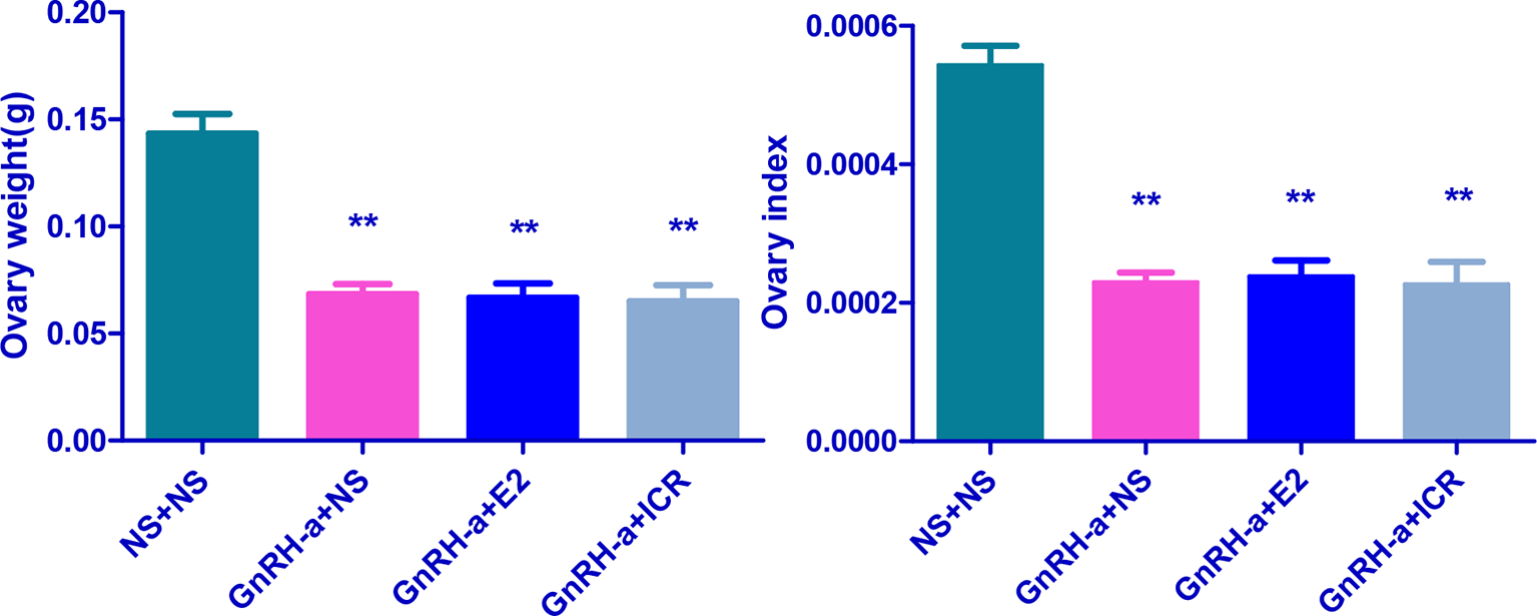
Comparison of ovarian weight and ovarian index of rats in each group after treatment. **P < 0.01 vs. the NS + NS group.

The ovarian weights of the NS + NS, GnRH-a + NS, GnRH-a + E2, and GnRH-a + ICR groups were (0.12 - 0.18)g, (0.06 - 0.08)g, (0.05 - 0.09)g, and (0.04 - 0.09)g, respectively. The ovarian indexes of rats in each group were (0.002164 ± 0.0004516), (0.0005123 ± 0.0001094), (0.001234 ± 0.0002007), and (0.0004177 ± 0.00001531), respectively. Compared with those in the NS + NS group, the ovarian weight and ovarian index in the GnRH-a + NS, GnRH-a + E2, and GnRH-a + ICR groups significantly decreased (P < 0.01). There was no statistical difference in the ovarian weight and ovarian index among the GnRH-a + E2, GnRH-a + NS, and GnRH-a + ICR groups (P > 0.05). See **Table 5** and **Figure 10**.

**Table 5:** Comparison of ovarian weight and ovarian index of rats in each group after treatment.

Group	N	Ovarian wet weight	Ovarian index
NS + NS	6	0.1433 ± 0.02251	0.002164 ± 0.0004516
GnRH-a + NS	6	0.06833 ± 0.01169**	0.0005123 ± 0.0001094**
GnRH-a + E2	6	0.06667 ± 0.01633**	0.001234 ± 0.0002007**
GnRH-a + ICR	6	0.0650 ± 0.01871**	0.0004177 ± 0.00001531**

**P < 0.01 vs. the NS + NS group.

#### Morphological Changes of Ovarian Tissues of Rats of Each Group

**Figure 11:**
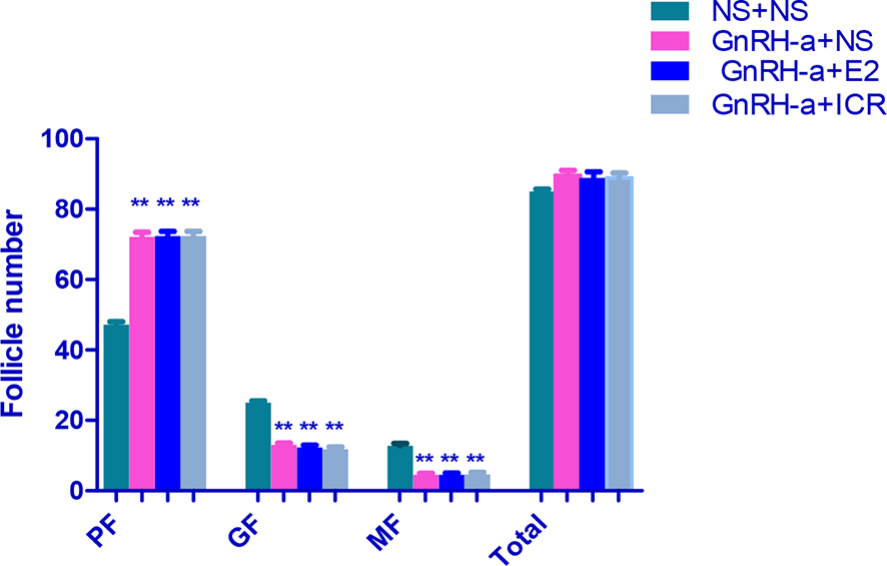
Comparison of number of follicles in bilateral ovaries of rats in each group. In the picture, PF, Primary Follicle; GF, Growing Follicle; MF, Mature Follicle. **P<0.01.

**Figure 12:**
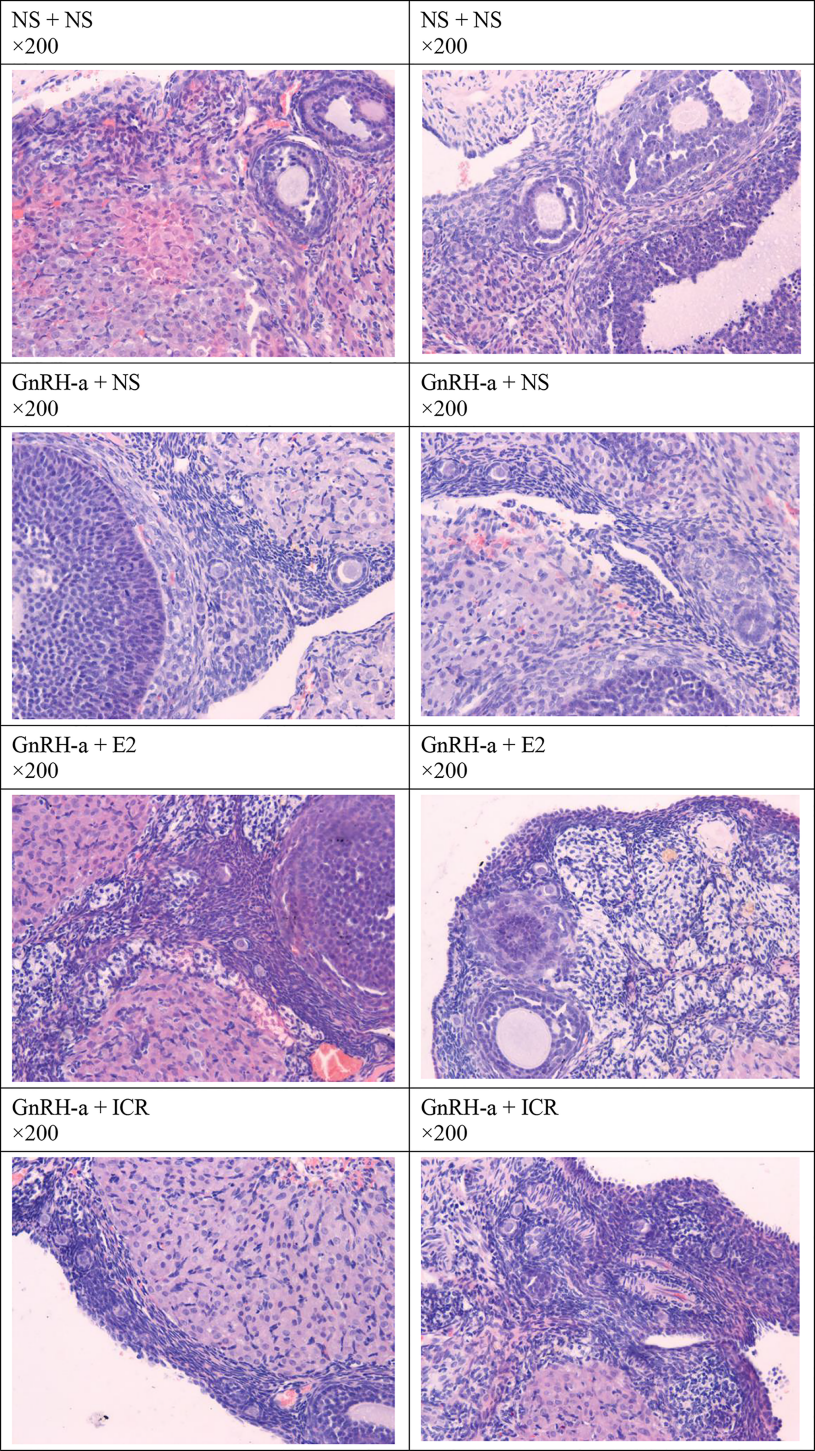
Histomorphological changes of bilateral ovaries of rats in each group.

The ovarian tissues of rats in each group were pathologically sectioned, stained with HE, and the morphological changes of ovarian tissues of each group were observed under microscope. The results showed that in the NS + NS group, the follicular structure was normal and all levels of follicles were visible. In the GnRH-a + NS group, the follicular structure was normal, the number of growing follicles and mature follicles decreased significantly, and the number of primordial follicles increased relatively. In the GnRH-a + E2 group, the follicular structure was normal, the number of growing follicles and mature follicles decreased significantly, and the number of primordial follicles increased relatively. In the GnRH-a + ICR group, the number of growing follicles and mature follicles decreased significantly, while the number of primordial follicles increased relatively. Compared with those in the NS + NS group, the number of primordial follicles increased significantly, while the number of growing follicles and mature follicles decreased significantly in the GnRH-a + NS, GnRH-a + E2, and GnRH-a + ICR groups (P < 0.01). There was no statistical difference in the total number of follicles among the four groups (P > 0.05). See **Table 6**, **Figures 11** and **12**.

**Table 6:** Comparison of number of follicles in bilateral ovaries of rats in each group.

Group	N	Primary Follicle	Growing Follicle	Mature Follicle	Total
NS + NS	6	47.17 ± 2.14	25.00 ± 1.41	12.83 ± 1.47	85.00 ± 1.79
GnRH-a + NS	6	72.17 ± 3.19**	13.00 ± 1.41**	4.50 ± 1.05**	89.67 ± 3.27
GnRH-a + E2	6	72.67 ± 3.72**	12.33 ± 1.51**	4.50 ± 1.38**	88.33 ± 5.54
GnRH-a + ICR	6	72.00 ± 2.97**	11.83 ± 1.47**	4.67 ± 1.37**	88.83 ± 3.55

**P < 0.01 vs. the NS + NS group.

## Discussion

Perimenopausal syndrome refers to a series of symptoms that seriously affect the quality of life of patients, such as hot flashes, sweating, irritability, insomnia, joint pain, and decreased sexual desire, which result from the decline, inhibition, or loss of the ovarian function caused by various reasons (16). Perimenopausal syndrome includes not only related low estrogen symptoms caused by the physiological ovarian function decline, but also the pathological ovarian function decline (premature ovarian failure) and iatrogenic ovarian function inhibition or loss, such as the destruction of the ovarian function by GnRH-a treatment or gynecological surgery (16, 17). EMS is a common gynecological benign disease, and its incidence in women of childbearing age is increasing year by year, which seriously affects the quality of life and fertility of patients (18). Surgical treatment is the main treatment method for EMS, but the recurrence after operation has been besetting clinicians (19). GnRH-a plays an important role in reducing the recurrence after EMS operation, and has become the most commonly used clinical method to prevent or delay the recurrence of EMS (20, 21). However, the perimenopausal symptoms such as hot flashes, vaginal dryness, loss of libido, and bone loss caused by the low estrogen level after the use of GnRH-a often restrict long-term and wide applications of GnRH-A. Although hormone-based add-back therapy can solve the perimenopausal symptoms well, long-term use of hormones may have risks (22, 23). Therefore, seeking other effective and safe add-back drugs is an issue demanding a prompt solution.

In order to evaluate the efficacy and safety of new drugs and study the mechanism of action of drugs, it is necessary to carry out animal experiments by establishing animal models reflecting corresponding disease states. Currently the replication of the pathological model of perimenopausal syndrome is mainly to cause ovarian failure by means of surgery and drug induction, such as castration (bilateral ovariectomy), X-ray injury, and ethanol injury to bilateral ovaries (24, 25). Moreover, animal models of natural aging can also be adopted. Specifically, the models of castrated rats and natural aging rats are the most widely used (26–28). The establishment of these models can reflect the characteristics of clinical perimenopausal syndrome from different aspects, and enable researchers to further understand and study the role of estrogen or other drugs in the perimenopausal and postmenopausal periods. Therefore, these animal models of perimenopausal syndrome established by removing or destroying ovaries in various ways play an irreplaceable role in the studies on evaluation and intervention of relevant perimenopausal symptoms. However, the animal model of perimenopausal syndrome established in this way and the animal model of perimenopausal symptoms caused by GnRH-a injection have the same physiological basis of a low estrogen status, but they cannot completely replace each other, and there are still big differences in the endocrine hormone status between them. The animal models of perimenopausal syndrome established by removing or destroying ovaries are low in estrogen. Moreover, due to the feedback mechanism of ovarian sex hormones to hypothalamus-pituitary-gonadal axis (HPOA), the serum FSH and LH levels of these models tend to increase obviously, that is, the estrogen deficiency in a high gonadotropin state. For the patients with perimenopausal symptoms caused by GnRH-a treatment after EMS, however, due to the down-regulation and desensitization of GnRH-a on the pituitary gland, the patients have not only a low estrogen level, but also significantly decreased FSH and LH levels, that is, estrogen deficiency in a low gonadotropin state, which is essentially different from the hormone physiological state of the animal model of perimenopausal syndrome mentioned above. Therefore, the rat model of perimenopausal symptoms built by using the GnRH-a injection to simulate GnRH-a treatment after EMS can better represent the hormone physiological state of patients with perimenopausal symptoms caused by GnRH-a treatment.

According to relevant literature (11, 13, 27), this study successfully established the rat model of perimenopausal symptoms by early injection of a relatively large dose of GnRH-a combined with maintenance with a small dose of GnRH-a. In order to identify whether modeling was successful, this study used the traditional vaginal smear method to observe the changes of vaginal cells in each group. The results showed that the vaginal cell smear of the control group showed obvious estrous cycle changes, while the rats in the GnRH-a injection model group had no estrous cycle of vaginal cells and stayed in the diestrus only, and the components of the cell smear were single. This fully indicates that the GnRH-a injection in this study successfully established the low estrogen status of rats. Furthermore, this study also observed that the temperature of the tail and anus of rats showed an upward trend after the GnRH-a injection, which indicated that after the GnRH-a injection, rats could have rising body surface temperature similar to that of hot flashes during the perimenopausal period, and the body surface temperature in the tail skin of rats increased earlier and more greatly than in the anus. This may be related to the manifestation of hot flashes in the perimenopausal period. Hot flashes are manifested as the expansion of blood vessels on the body surface and the increase of heat dissipation. Therefore, it is speculated that the tail surface temperature of the rats may be more representative of the occurrence of hot flashes in the perimenopausal period, which complies with the viewpoints of Kobayashi et al. (29).

The trial results of this study also showed that GnRH-a injection could significantly facilitate the weight increase trend of rats, significantly decrease the serum E2, FSH, and LH levels, decrease the uterine weight, uterine index, ovarian weight, and ovarian index, significantly reduce the number of growing follicles and mature follicles, and increase the proportion of primordial follicles. These results fully indicate that GnRH-a injection could successfully construct the rat model of perimenopausal symptoms, and well represent the hormone status of low E2, low FSH, and low LH levels and the physiological changes such as weight gain after low estrogen in patients with perimenopausal symptoms caused by clinical GnRH-a treatment. Furthermore, the results of this study also showed that the changes of body temperature and weight gain of the perimenopausal rat model in this group were significantly improved after the intervention with estradiol and black cohosh preparations, which indicated that estradiol and black cohosh preparations could effectively alleviate or relieve the perimenopausal symptoms and physiological state of weight gain caused by GnRH-A. For the black cohosh preparation group, the serum E2 level, uterus weight, uterus index, ovary weight, and ovary index of rats were not different from those of the control group after intervention, and were significantly lower than those in the estradiol intervention group. This indicates that black cohosh preparations had no obvious estrogen-like effect, and its effect of relieving the perimenopausal symptoms was not directly exerted by estrogen, possibly through other mechanisms, which will be specifically discussed in the next part of this study.

In conclusion, the rat model of perimenopausal symptoms could be successfully built through the GnRH-a injection. The animal model successfully achieved significant decreases of the E2, FSH, and LH levels in the rats, and could cause the rats to have a rising body surface temperature similar to perimenopausal hot flashes, which was more in line with the hormone physiological state of patients with perimenopausal symptoms caused by GnRH-a treatment in clinical practice. The intervention with estradiol and black cohosh preparations could effectively improve such “perimenopausal state”. Moreover, black cohosh preparations had no obvious effect on the serum sex hormone level of the rat model, and had no obvious estrogen-like effect. Its mechanism of exerting clinical effects needs to be further studied.

## Data Availability Statement

The original contributions presented in the study are included in the article/supplementary material. Further inquiries can be directed to the corresponding authors.

## Ethics Statement

The animal study was reviewed and approved by The First Clinical College of Nanjing Medical University.

## Author Contributions

All authors listed have made a substantial, direct, and intellectual contribution to the work, and approved it for publication.

## Funding

This work was supported by grants from the Scientific Research Support Program for Postdoctoral of Jiangsu Province (2019K064), the Major Science and Technology Program of Changzhou Health and Family Planning Commission (ZD201812), and the Scientific Research Support Program for “333 Project” of Jiangsu Province (BRA2019161).

## Conflict of Interest

The authors declare that the research was conducted in the absence of any commercial or financial relationships that could be construed as a potential conflict of interest.

## Publisher’s Note

All claims expressed in this article are solely those of the authors and do not necessarily represent those of their affiliated organizations, or those of the publisher, the editors and the reviewers. Any product that may be evaluated in this article, or claim that may be made by its manufacturer, is not guaranteed or endorsed by the publisher.
